# Levels of soluble VCAM-1, soluble ICAM-1, and soluble E-selectin in patients with tuberculous pleuritis

**DOI:** 10.1155/S0962935196000403

**Published:** 1996-09

**Authors:** A. Hamzaoui, K. Hamzaoui, A. Kahan, A. Chabbou

**Affiliations:** 1 Institute of Chest Diseases Ariana Tunisia; 2 Medical University of Tunis 9, Rue Z. Essafi Tunis 1007 Tunisia; 3 INSERM U.283 Cochin Hospital Paris France

## Abstract

Tuberculosis is characterized by the presence of activated mononuclear cells both in the peripheral circulation and in pleural fluid. Expression and up-regulation of adhesion molecules is the basis of cell-cell adhesion in granuloma formation and in leukocyte migration to the inflammatory site. Soluble isoforms of adhesion molecules have been described, and their expression at high levels indicated an activated state. The purpose of this study was to evaluate levels of soluble adhesion molecules in serum and pleural fluid from patients with tuberculous pleural effusions, compared with non-tuberculous pleural effusions. We analysed levels of soluble vascular cell adhesion molecule-1 (s.VCAM-1), soluble intercellular adhesion molecule-1 (s.ICAM-1), and soluble E-selectin (sE-selectin) in serum and pleural fluid from patients with tuberculous pleuritis, by sandwich ELISA. Serum levels of s.ICAM-1 and s.VCAM-1 in patients with tuberculosis were higher than those in healthy controls (p < 0.001). Levels of sE-selectin levels were in the normal range compared with control groups. In pleural fluid, levels of s.VCAM-1 and s.ICAM-1 were increased in pleural effusions. Patients with tuberculous pleural effusion exhibited high levels of s.ICAM-1 compared with patients with neoplastic pleural involvement. Up-regulation of s.VCAM-1 and s.ICAM-1 in serum, along with increased levels of sE-selectin in pleural effusions from tuberculous patients, may result in transmigration of activated inflammatory cells inducing pleural damage, which may contribute to the pathological processes involved.


Mediators of Inflammation 5, 276–279 (1996)

### Levels of soluble VCAM-1, soluble ICAM-1, and soluble E-selectin in patients with tuberculous pleuritis

A. Hamzaoui,[1] K. Hamzaoui,[2,CA] A. Kahan[3] and A. Chabbou[1]

[1]Institute of Chest Diseases, Ariana, Tunisia;
[2]Medical University of Tunis, 9, Rue Z. Essafi, 1007 Tunis, Tunisia;
[3]INSERM U.283, Cochin Hospital, Paris, France

[CA]Corresponding Author

**Tuberculosis is characterized by the presence of activated mononuclear cells both in the peripheral circulation and in pleural fluid. Expression and up-regulation of adhesion molecules is the basis of cell–cell adhesion in granuloma formation and in leukocyte migration to the inflammatory site. Soluble isoforms of adhesion molecules have been described, and their expression at high levels indicated an activated state. The purpose of this study was to evaluate levels of soluble adhesion molecules in serum and pleural fluid from patients with tuberculous pleural effusions, compared with non-tuberculous pleural effusions. We analysed levels of soluble vascular cell adhesion molecule-1 (s.VCAM-1), soluble intercellular adhesion molecule-1 (s.ICAM-1), and soluble E-selectin (sE-selectin) in serum and pleural fluid from patients with tuberculous pleuritis, by sandwich ELISA. Serum levels of s.ICAM-1 and s.VCAM-1 in patients with tuberculosis were higher than those in healthy controls ($p < 0.001$). Levels of sE-selectin levels were in the normal range compared with control groups. In pleural fluid, levels of s.VCAM-1 and s.ICAM-1 were increased in pleural effusions. Patients with tuberculous pleural effusion exhibited high levels of s.ICAM-1 compared with patients with neoplastic pleural involvement. Up-regulation of s.VCAM-1 and s.ICAM-1 in serum, along with increased levels of sE-selectin in pleural effusions from tuberculous patients, may result in transmigration of activated inflammatory cells inducing pleural damage, which may contribute to the pathological processes involved.**

**Key words:** Lung, *Mycobacterium tuberculosis*, Soluble adhesion molecules

### Introduction

Infection with *Mycobacterium tuberculosis* leads to the activation of macrophages and lymphocytes and to the formation of granuloma.[1] The immunopathologic status of tuberculous pleuritis is characterized by a number of T-lymphocyte abnormalities.[2] Lymphocytosis in tuberculosis infection involves both CD4 and CD8 cells. The pleural effusion cells contain T-lymphocytes, with an inverted CD45RA to CD45RO ratio, and a high level of interleukin-2 receptor.[3] The sites of tuberculosis inflammation are characterized by Th1 cytokines: interferon-gamma, IL-2 product, significantly greater than corresponding concentrations in supernatants of stimulated peripheral blood T-lymphocytes.[4] Accumulation of monocytes, lymphocytes and polymorphs occurs at the sites of infection following cell adhesion to endothelial cells and migration from blood vessels into tissues.[5] These adhesive processes are mediated by a variety of adhesion proteins: adhesion molecules, which play key roles in the pathogenesis of tuberculosis.[6] *Mycobacterium tuberculosis*, the causative agent of tuberculosis, is an obligate intracellular pathogen and was the causative agent of activation of mononuclear cells both in the peripheral circulation and in pleural fluid from tuberculous patients.[6]

*In vitro* studies with blocking antibodies have identified adhesion molecules participating in the adherence of macrophages to one another, to T-lymphocytes and to vascular endothelial cells. E-selectin is expressed exclusively on endothelial cells after stimulation with cytokines *in vitro*.[7] It interacts with carbohydrate structures on circulating cells, thereby initiating the so-called 'rolling' of leukocytes on the endothelial surface[7] with subsequent up-regulation of the expression of intercellular adhesion molecule-1 (ICAM-1) and

© 1996 Rapid Science Publishers vascular cell adhesion molecule-1 (VCAM-1) on the endothelial surface. Cell activation results in the shedding of adhesion molecules, which may then be detected in a soluble form in serum. Soluble isoforms of adhesion molecules: ICAM-1, VCAM-1, and E-selectin are capable of binding to their respective ligands after stimulation of endothelial cells with cytokines *in vitro*.[8] Levels of these isoforms may reflect enhanced expression of adhesion molecules on immunocompetent activated cells. To investigate the involvement of these molecules, we measured levels of soluble VCAM-1 (s.VCAM-1), soluble ICAM-1 (s.ICAM-1), and soluble E-selectin (sE-selectin) in sera and in pleural fluid effusion from tuberculous patients.

### Materials and Methods

*Patients:* This study concerns 25 patients with pleural effusions. The differential diagnosis between the two clinical situations studied (tuberculous and neoplastic) was based on other laboratories tests (protein, amylase, pH values, etc.), on the presence on direct examination and positive cultures of *Mycobacterium tuberculosis*, on the histopathology of pleural biopsies, and on a search for malignant cells in the pleural fluid. This method allowed the formation of two groups: (A) 15 patients with tuberculous pleural effusions, with an average age of 34 $\pm$ 5.4 years; and (B) ten patients with neoplastic pleural effusions, with an average age of 58.3 $\pm$ 10.8 years.

*Pleural exudates:* Soluble vascular cell adhesion molecule-1 (s.VCAM-1), soluble intercellular adhesion molecule-1 (s.ICAM-1), and sE-selectin, were studied in pleural exudates and in serum obtained from 25 patients (15 patients with tuberculous pleural effusions and ten patients with neoplastic pleural effusions). Sera from 20 healthy controls with an average age of 36 $\pm$ 7.2 years were also studied.

*s.VCAM-1, s.ICAM-1 and sE-selectin analysis:* Serum and pleural fluids levels of s.VCAM-1, s.ICAM-1 and sE-selectin were analysed by sandwich ELISA according to the instructions of the manufacturer (British Biotechnology Products Ltd, Abingdon, UK). All measurements were done with the same batch in duplicate. In brief, microtitre plates were coated with a murine IgG class monoclonal antibody (MoAb) directed against one epitope on s.VCAM-1, s.ICAM-1 or sE-selectin, respectively. After incubation with serum samples or standards in appropriate dilution, a biotinylated murine IgG class MoAb directed against a second epitope on s.VCAM-1, s.ICAM-1 and sE-selectin, respectively, was added. After addition of streptavidin conjugated horseradish peroxidase (HRP), colour reaction was obtained by tetramethylbenzidine, and the plates were read on an automated multiscanner. To determine the normal range, serum control samples were obtained from 20 healthy individuals. The mean $\pm$ 2 SD from this control group was taken as the normal range.

*Stastistical analysis:* All measurements are shown as the mean $\pm$ SD. In the statistical analysis of the data, we used the Student–Fisher *t*-test.

### Results

*Level of soluble adhesion molecules in serum:* The mean serum values in 20 healthy individuals ($\pm$ SD) for s.VCAM-1, s.ICAM-1 and sE-selectin were 438 $\pm$ 140 ng/ml, 329 $\pm$ 56 ng/ml and 53 $\pm$ 17 ng/ml, respectively. Soluble serum VCAM-1 (620 $\pm$ 56 ng/ml) and s.ICAM-1 (536 $\pm$ 23 ng/ml), exhibited increased levels in patients with tuberculosis, compared with healthy controls ($p < 0.01$). Levels of sE-selectin in serum from tuberculosis patients (60 $\pm$ 19 ng/ml) were not different from those of controls.

In serum from patients with neoplastic pleural involvement, s.VCAM-1 (630 $\pm$ 180 ng/ml) and s.ICAM-1 (430 $\pm$ 32 ng/ml) were higher than healthy controls ($p < 0.001$). Soluble E-selectin

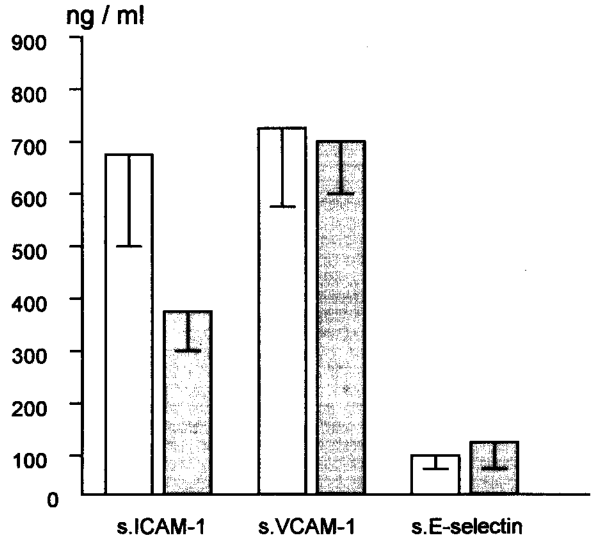

FIG. 1. Pleural fluid levels of soluble vascular cell adhesion molecule-1 (s.VCAM-1), soluble intercellular molecule-1 (s.ICAM-1), and sE-selectin in 15 patients with tuberculous pleural effusions □ and ten patients with neoplastic effusions ▒. Concentrations are given as ng/ml. Levels of s.ICAM-1 are significantly higher in patients with pleural tuberculosis ($p < 0.001$), and s.VCAM-1 and E-selectin are compatible with patients with neoplastic effusions.

were at the normal range (58 ± 22 ng/ml) as for healthy controls. A significant difference was found in s.ICAM-1 levels between patients with tuberculosis and patients with neoplastic pleural fluid ($p < 0.01$).

*Level of soluble adhesion molecules in pleural effusions:* Tuberculous and nontuberculous pleural effusions exhibited similar levels of s.VCAM-1 and sE-selectin (Fig. 1). Soluble ICAM-1 levels were higher in patients with tuberculosis (672 ± 31 ng/ml) compared with patients with neoplastic pleural involvement (390 ± 61 ng/ml; $p < 0.001$). No correlation was found between the levels of the three adhesion molecules studied both in serum and in pleural effusions.

### Discussion

In this study, we analysed levels of soluble adhesion molecules in serum and in pleural effusion in patients with tuberculosis, to analyse the state of activation of the immune system in the site of inflammation. Both in serum and in pleural fluid, patients with tuberculosis were characterized by increased levels of s.VCAM-1 and s.ICAM-1. In their pleural effusion, patients with tuberculosis were characterized by the high level of s.ICAM-1, compared with neoplastic pleural effusion.

The host response to *M. tuberculosis* is characterized by interactions between mononuclear cells, with recruitment and fusion of these cells cumulating in granuloma formation. This response requires $CD4^+$ T-cell reactivity, mediated by antigen-independent as well as antigen-dependent mechanisms. A continuous upregulation of the expression of ICAM-1 on mononuclear phagocytes induced by *M. tuberculosis* was reported.[9–11] The increase in ICAM-1 protein expression was accompanied by an increase in steady-state mRNA analysis,[9] but neither the expression of VCAM-1 nor CD11a/CD18 or CD11b/CD18 were increased to a similar extent. Circulating ICAM-1, VCAM-1 and E-selectin were found to be elevated in serum patients with tuberculosis.[10] Our data support these findings, plus an increased expression of ICAM-1 in pleural fluid effusion. The release of ICAM-1 and VCAM-1 in serum and pleural fluid may indicate their overexpression. Important immunopathological consequences of experimental mycobacterial infection is the suppression of T-cell-mediated immune response to both mitogens and mycobacterial antigens. Upon infection with *M. tuberculosis* ICAM-1 expression on infected macrophages was increased in BALB/c mice.[12] The changes of ICAM-1 expression on the surface of infected macrophages resulted in inhibition of delayed-type hypersensitivity-mediating functions of T-helper cells from BALB-c mice. The presence of soluble adhesion molecules in serum and in pleural fluid effusion, support that this could be a pathway of specific susceptibility to *M. tuberculosis* infection.[12]

Activation of the endothelium may result in increased expression of adhesion molecules. Immunohistological examination of the tuberculous lung tissues from patients showed intensive cellular ICAM-1 expression on epitheloid cells and giant cells as well as on vascular endothelial cells.[13] Levels of the soluble forms of the more broadly expressed ICAM-1 and VCAM-1 were significantly elevated in sera whereas those of E-selectin, which is only expressed on endothelial cells, were not. Tuberculous and nontuberculous pleural effusion exhibited similar levels of s.VCAM-1 and sE-selectin, contrasting with a significant difference in s.ICAM-1 expression. The differential local expression of adhesion molecules may reflect differences in local cytokine concentrations. The expression of E-selectin, VCAM-1 and ICAM-1 on endothelial cells is induced by IL-1 and TNF-α, both of which are produced in increased amounts in tuberculosis[4] and neoplastic pleural effusions.[14] Thus, *in vitro* T-cell clones specific for purified protein derivative of *Mycobacterium tuberculosis* have the same cytokine secretory profile as TH1 cells, producing IL-1, TNF-α, IFN-γ.[10] *In vitro* and animal studies have suggested differential endothelial expression of ICAM-1, VCAM-1 and E-selectin upon stimulation with different combinations of IL-1, TNF-α and IL-4.[15]

At the present time the significance of release of these molecules is unknown. The physiological importance of these soluble molecules is unclear, but the presence of soluble adhesion molecules may influence T-lymphocyte function. Since they retain the capacity to bound to their ligands, they may regulate cell adhesion by competition,[16] or trigger a response in a ligand-bearing cell. Tuberculous pleural effusions are characterized by a lymphocytosis containing a majority of T $CD4^+$ lymphocytes. These lymphocytes show an inverted CD45RA to CD45RO ratio, and high levels of the IL-2 receptor in some patients, suggesting the existence of periods of cell activation together with non-activation periods.[3] Soluble adhesion molecules may be involved in modulating T-cell activation. Further studies are needed to clarify the precise pathophysiological role of s.VCAM-1 and s.ICAM-1 in tuberculosis.

### References

1. Edwards D, Kirkpatrick C. The immunology of mycobacterial diseases. *Am Rev Respir Dis* 1986; **134:** 1062–1071.
2. Arzabe RA, Machado IV, Fernandez B, Blanca I, Ramirez R, Bianco NE. Cellular immunity in current active pulmonary tuberculosis. *Am Rev Resp Dis* 1991; **14:** 496–500.
3. Gambon D, Pacheo-Carracedo M, Cedra-Mota T, Montes-Santiago J. Lymphocytes population during tuberculosis infection: V beta repertories. *Infect Immun* 1995; **63:** 1235–1240.
4. Barnes PF, Lu-S, Abranis JS, Wang E, Yamamura M, Modlin RL. Cytokine production at the site of disease in human tuberculosis. *Infect Immun* 1993; **61:** 3482–3489.
5. Ozaki T, Nakahira S, Tank K, Ogushi F, Yasuoka S, Ogura T. Differential cell analysis in bronchoalveolar lavage fluid from pulmonary lesions of patients with tuberculosis. *Chest* 1992; **102:** 54–59.
6. Yassin RJ, Hamblin AS. Altered expression of CD11/CD18 on the peripheral blood phagocytes of patients with tuberculosis. *Clin Exp Immunol* 1994; **97:** 120–125.
7. Sfifakis PP, Tsokos GC. Lymphocyte adhesion molecules in autoimmune rheumatic diseases: basic and clinical expectations. *Clin Exp Rheumatol* 1995; **13:** 763–778.
8. Wellicome SM, Kapahi P, Mason JC, Lebranchu Y, Yarwood H, Haskard DO. Detection of a circulating form of vascular cell adhesion molecule-1: raised levels in rheumatoid arthritis and systemic lupus erythematosus. *Clin Exp Immunol* 1993; **92:** 412–418.
9. Lopez-Ramirez GM, Rom WN, Ciotoli C, Talbot A, Martiniuk F, Cronstein B, Reibman J. *Mycobacterium tuberculosis* alters expression of adhesion molecule on monocytic cells. *Infect Immun* 1994; **62:** 2515–2520.
10. Lai CK, Wong KC, Chan CH, Ho SS, Chung SY, Haskard DO, Lai KN. Circulating adhesion molecules in tuberculosis. *Clin Exp Immunol* 1993; **3:** 522–526.
11. Shijubo N, Imai K, Nakanishi A, Abe S. Elevated concentrations of circulating ICAM-1 in far advanced and miliary tuberculosis. *Am Rev Respir Dis* 1993; **148:** 1298–1301.
12. Saha B, Das G, Vohra H, Ganguly NK, Mishra GC. Macrophage T cell interaction in experimental mycobacterial infection. Selective regulation of co-stimulatory molecules on Mycobacterium-infected macrophages and its implication in the suppression of cell-mediated immune response. *Eur J Immunol* 1994; **24:** 2618–2624.
13. Shijubo N, Hirasawa M, Inuzuka M, Asakawa M, Abe S, Imai K. Intercellular adhesion molecule 1 antigen in tuberculosis. *Kekkaku* 1994; **69:** 471–474.
14. Gjomarkaj M, Pace E, Melis M, Spatafora M, Toews GB. Mononuclear cells in exudative malignant pleural effusions. Characterization of pleural phagocytic cells. *Chest* 1994; **106:** 1042–1049.
15. Stegeman AC, Cohen Tervaert WJ, Huitema MG, de Jong PE, Kallenberg GM. Serum levels of soluble adhesion molecules intercellular adhesion molecule-1, vascular cell adhesion molecule 1, and E-selectin in patients with Wegener's granulomatosis. *Arthritis Rheum* 1994; **37:** 1228–1235.
16. Jones SC, Banks RE, Haidar A, Gearing AJH, Hemingways IK, Ibbotson SH, Dixon MF, Axon ATR. Adhesion molecules in inflammatory bowel disease. *Gut* 1995; **36:** 724–730.

ACKNOWLEDGEMENT. This work was supported by the Ministry of Education and Science of Tunisia.

**Received 23 May 1996; accepted in revised form 2 July 1996**
